# Dental Laws

**Published:** 1868-11

**Authors:** B. T. Whitney

**Affiliations:** Buffalo, N. Y.


### DENTAL LAWS.

Dr. Taft. Dear Sir.—As there is, just now, considerable interest felt in the matter of Dental laws, and some criticism on those passed by the legislatures of a few of the States and Canada, I ask a little space in the Register to give some explanation as to the features of that of New York; and why it is so moderate, and apparently impotant in securing the needed reform at once—in weeding out the incompetent, and securing a higher standard of qualification. And public reformation, like individual education, must have some starting point. Now, it is proposed to take the present state of Dentistry as this starting point; and we hope to make rapid progress.

From the known liberality of the great mass of the people and especially of the legislative body, in all matters of franchise or business, it was believed that a law stringent enough to secure all that might be desired in regulating the practice of Dentistry, could not be carried through our legislature; and even if it could, doubtless some one feeling himself aggrieved, would test its constitutionality through the courts.

In either case we should, if not loose all, at least thereby create a prejudice that could not be easily overcome. It was evident that if we did not ask too much, we could promptly obtain all that is now granted to the medical profession; and thus secure a legal status, with society organizations. With this machinery we could gain influence, and grow in favor with the public, and soon educate them and the Dental profession for greater reforms; and thus go on, step by step, as the child learns to walk, until all that laws can do for us, may be obtained.

With this view of the case, it seemed that any law must be prospective in its operations, and not retrospective—that we could not reach those that were already established in practice, however moderate their ability; but could ultimately fix a certain standard for those who come into the profession hereafter. It is entirely competent for the legislature to fix by law certain conditions, or certain qualifications for those who are to commence any business; but not to proscribe those already engaged in it—not to erect a guillotine for the incompetent. The constitution and laws of New York give to every person a right to engage in any branch of business, not otherwise provided for or guarded against, and not legally obnoxious, without any specific legal qualifications or legal restraints; and the State legislature has no power to legislate away their rights or established business. Such a law, with us, would not only be considered oppressive, but unconstitutional, and the Court of Appeals would so decide.

The position of the Dental profession is very much like that of the medical in this State previous to legislative protection; and as we had a precedent in her, we thought it best to follow her maternal teachings and be satisfied.

The first general laws for the formation of medical societies, and regulating the practice of medicine and surgery in the State of New York, were passed on the 4th day of April, 1806. By subsequent amendments and additions up to 1813, there was secured to the profession, and the people, probably the best code of medical laws and society organizations of any State in the Union. Every clause in these laws that could be construed as in the least exclusive or restrictive in its operation, was contended against and modified, but carried through by dint of earnest effort and perseverance for the public good. All went on well under these laws for about a generation; when the various forms of quackery sprang up, and gained some foothold with the public. Their advocates claimed that as they had moral and religious rights, they should also have legal rights.

The Root Doctor, the Thompsonian, and the Hydropathics made common cause against the manopoly given to the regular medical profession—against *mineral poisons*, declaring that in the products of our native soil there were abundant medical agents to cure the diseases of our climate. Finally, the Homeopathic creed put forward its sugar pills and infinitesimal doses, which so tickled the fancy, and pleased the palate of a portion of the people, that they joined in the clamor. The legislature was besieged for several years, and finally, in 1844 the fatal blow was struck, and all laws restricting the practice of medicine, and even those relating to the qualification of persons entering the profession were repealed, leaving it open alike to all who choose to engage in the business. The laws knew no difference between the most thoroughly educated practitioner, and the most arrant quack, or feeble Herb Doctor. There are but two features of that noble old law left that are valuable, that of the society organiz itions; and, in case of judgment for malpractice, criminal proceedings might be had against the irregular practitioner, but not against those of the regular school and members of the legal societies. Those societies are for each county, and a State society, which is made up of delegates from the several county societies and medical colleges in this State, and permanent members who are elected from the delegates who have served their term as such. As the number of Dentists would make the organizations small in most of the counties, the

State was divided into eight districts, taking the present Supreme Judicial Districts as the best division. The State Dental Society is made up of delegates, &c., in the same manner as the State Medical Society. These are the only Dental societies in this State under the sanction of this law; but these do not, in any way, effect the several local voluntary associations for mutual improvement. But these voluntary societies can have no representation in the State Dental Society.

As the same objections do not hold against the Dental as against medical laws—there being no conflicting systems or modes of practice—no "*'pathies*" to covet favor, there can be no organized or very strong opposition brought to bear upon the legislature; and moreover the public will soon ask farther protection.

We really feel that, in New York to-day, our position is stronger than those who have taken a longer stride. The first step is a moderate one, but one that will naturally carry us forward in the right direction, without any fear of retrograding. We apprehend that in a year or two, there will be no obstacles to our obtaining as strict laws as in any other State, or that can be desired, or that may be for the good of all. No muddy stream can be immediately cleansed. The impurities will gradually settle to the bottom, or be carried on with the current into the ocean, and pure water flow in its place.

I presume that most of the States have constitutional provisions, laws and customs, much like ours in New York, which may be in the way of very stringent Dental laws; but a moderate and prospective law might easily be obtained in any State where a more stringent one cannot; and thus give Dentistry a legal recognition and a status—a firm foundation to stand upon. It could then be easily followed up with such amendments as the people, the profession and the legislature may be prepared for. B. T. WHITNEY.

BUFFALO, N. Y., Aug. 20, 1868.

